# Survey on the resolution and accuracy of input data validity for SWAT-based hydrological models

**DOI:** 10.1016/j.heliyon.2024.e38348

**Published:** 2024-09-24

**Authors:** Nisreen Jawad Rasheed, Mahmoud S. Al-Khafaji, Imzahim A. Alwan, Mohammad Saleh Al-Suwaiyan, Ziaul Haq Doost, Zaher Mundher Yaseen

**Affiliations:** a Soil and Water Resources Department, College of Agriculture, University of Diyala, Baqubah, 32001, Diyala, Iraq; b Department of Water Resources Engineering, University of Baghdad, Baghdad, Iraq; c Civil Engineering Department, University of Technology, Baghdad, 10066, Iraq; d Civil and Environmental Engineering Department, King Fahd University of Petroleum & Minerals, Dhahran 31261, Saudi Arabia; e Interdisciplinary Research Center for Construction and Building Materials, King Fahd University of Petroleum & Minerals (KFUPM), 31261, Dhahran, Saudi Arabia; f Interdisciplinary Research Center for Membranes and Water Security, King Fahd University of Petroleum & Minerals, Dhahran, 31261, Saudi Arabia

**Keywords:** SWAT, Spatial resolution, DEM resolution, Soil map resolution, LULC resolution, Alternative climate products

## Abstract

This review was conducted to highlight the most influential factors and specify the trends reducing uncertainty and increasing the accuracy of soil and water assessment tool (SWAT)-based hydrological models. Although the resolution of input data on the results of SWAT-based hydrological models has been extensively determined. There is still a gap in providing comprehensive review framework to be emerged for identifying the impact of the data resolution and accuracy. The factors taken into consideration in this study were the impact of digital elevation model (DEM) resolution, soil data resolution, land use and land cover (LULC) resolution, and the impact of weather data resolution. Identifying the best DEM resolution depends on the watershed response and hydrological processes. However, for sediment yield estimation, more attention should be paid to the accuracy of soil data. Furthermore, the impact of LULC resolution on the accuracy of streamflow is still not sufficiently understood, whereas fine resolution is required for an accurate simulation of the sediment yield. Sub-daily precipitation data is essential for an accurate estimation of streamflow. Despite the fact that climate forecast system reanalysis (CFSR) and tropical rainfall measuring mission (TRMM) are the most widely used climate products, climate hazards group infrared precipitation with station data (CHIRPS) produces an adequate estimation for streamflow when there is insufficient gauged data. However, other aspects have not been deeply taken into consideration, including the interactive and complementary impacts of these factors. Thus, more attention and focus should be given to these issues. This review and evaluation can be a significant guide for selecting the suitable input data to implement efficient SWAT-based watershed models.

## 1 Introduction

### 1.1 Research background

Hydrological models are generally used to predict how hydrological processes and/or water quality processes will respond to changes in environmental and human factors as well as to various management approaches [[1], [2], [3]]. Identification of reliable hydrological modeling tools is an important issue for local authorities in coming up with effective solutions to the problems of floods, droughts, and pollutant exports [4,5]. Yet, hydrological and climate models as well as the resolution of input data are all major sources of uncertainty currently found in hydrological modeling frameworks [5,6]. Consequently, ongoing attempts to enhance modeling approaches by addressing these difficulties. In addressing the challenges of sustainable water management, a research emphasized the critical role of multi-criteria analysis in selecting dam sites, which directly impacts the effectiveness of runoff management strategies essential for maintaining water sustainability [7]. In hydrological studies, the application of appropriate data and practical models is necessary to comprehend basin-scale hydrological processes [8]. An important source of uncertainty in hydrological modeling predictions is the accuracy and quality of the input data, which in turn affects hydrological models' capacity to calculate variables like surface runoff, sediment yield, and nutrients in a watershed [9]. In recent years, many studies have been conducted on the simulation of hydrological models, particularly rainfall-runoff and flood prediction models [[10], [11], [12]].

A globally frequently used hydrological modeling tool is SWAT [[13], [14], [15]]. In addition, more than 4300 SWAT-related articles have been published [3], where, from a list of over 70 models, SWAT is the most widely used model for simulating streamflow and sediment yield [16]. The ability of SWAT to accurately predict streamflow and sediment yield heavily depends on the quality of the Digital Elevation Model (DEM), soil data, land use and land cover (LULC), and weather data as input variables. One of the fundamental input files for the SWAT is the DEM file, which is used in the SWAT and many other hydrological models for watershed delineation [[17], [18], [19]]. The uncertainties in the values of streamflow and sediment yield due to the change in DEM resolutions were identified in this study. Furthermore, appropriate soil data is a crucial input for the SWAT. The relevance of these data increased as the scale of the study decreased. Moreover, the LULC data is one of the most critical elements impacting watershed hydrology. The spatial and temporal variability of precipitation is the primary source of uncertainty in SWAT simulation and flood forecasting [[20], [21], [22]].

Precipitation, on the other hand, is a critical input parameter for hydrological models [[23], [24], [25], [26]] and the most influential and active factor in studies of atmospheric climate [27]. Reliable and accurate precipitation data is required to effectively manage water resources and accurately predict the weather, hydrology, and climate data [[28], [29], [30]]. Precipitation may be measured directly at the Earth's surface using gauge observations [31]. However, gauge observations of precipitation datasets may have incompleteness, missing values, inhomogeneous issues, and sparse distribution [[32], [33], [34], [35]], as well as long-term precipitation data may be inaccessible or unusable in some nations due to the lack of resources, political instability, disinterest, and other factors. Therefore, alternative climate data sources, including satellite precipitation, gauge-based gridded data, reanalysis products, and ground-based weather radar, can produce a reliable result, notably in the case of limited or ungauged river systems [3,36].

Often, modelers are forced to strike a balance between computation time and accuracy, depending on the model application's purpose. Using high spatial resolution data can extend computation time and is often impractical when modeling large watersheds [37]. Calibrating models with high spatial or temporal resolution might take much time [38]. Due to the resource and financial constraints faced by many watershed managers, particularly where watersheds span administrative or political borders [39], it would be beneficial to identify the appropriate spatial and temporal resolution that gets the best accuracy for runoff and sediment yield in watershed modeling. In other words, the right spatial and temporal resolution is needed to deal with the lack of data, time, and resource constraints [37,38].

### 1.2 The existed review research on SWAT-based hydrological models

In the light of existed literature review, there are several studies conducted on reviewing the application of SWAT hydrological model. In 2014, a comprehensive analysis was conducted to evaluate utilization of the SWAT-hydrological model [40]. As reported in the review study, the investigation encompassed a meticulous examination of 22 scholarly inquiries spanning four principal categories, namely scenario analysis, hydrologic foundation, sediment transport and routing analysis, and nutrient and pesticide transport to scrutinize the practicality and effectiveness of the SWAT model [40]. The overarching findings of this extensive overview demonstrate that the SWAT model serves as an exceptional tool for various water resources and land management applications. Additionally, the study revealed certain deficiencies inherent in the SWAT model, particularly concerning input data, which necessitate specific enhancements and a broader spectrum of testing [40]. Furthermore, in 2017, a comprehensive research study was conducted, employing a survey-based approach, to assess the repercussions of input data scale on the outcomes generated by modeling processes [41]. The outcomes of the review research establish a crucial connection between the input and output data, emphasizing the necessity to scrutinize the output at each scale of input data to ascertain the optimal output results [41]. Remarkably, the study reveals that input DEM files with finer resolutions yield more precise output results, while the impact of LULC on the output data remains ambiguous [41]. Similarly, a study demonstrated that changes in LULC significantly influenced groundwater fluctuations, providing clear evidence of how LULC dynamics can affect hydrological outcomes [42]. As a result, the research underscores the imperative to investigate resolution of the input data including climatological factors for a more comprehensive analysis of the model [41].

Moreover, in the year 2023, a comprehensive academic review study was undertaken to assess the strengths and weaknesses of the SWAT model in hydrological process modeling [43]. The research unequivocally demonstrated that the SWAT model constitutes a highly robust and freely available tool for scientists engaging in watershed modeling endeavors [43]. Notably, the SWAT model possesses the capability to simulate human activities and agricultural interventions [43,44]. Nevertheless, it is imperative to acknowledge certain shortcomings within the hydrological application of the SWAT model, particularly with regard to the meticulous examination of temporal data [43]. The model necessitates an extensive array of data inputs, encompassing topographical details, soil type and characteristics, land cover information, flow data, meteorological data, sediment data, nutrient data, and more [43]. Therefore, it is crucial to investigate the impacts of resolution of input data and their subsequent calibration processes on the output results [43]. In the same way, in 2023 a review article, investigated the application, feasibility, and future work of SWAT model on Mediterranean catchments [45]. The study covered 260 academic studies in the Mediterranean catchments to investigate the feasibility of the SWAT model [45]. The review research discovered that the examined articles exhibited diverse applications of SWAT in several categories [45]. These encompassed the evaluation of water resource quality and quantity, the foundational assessment of model performance, the prediction of hydrological and pollutant losses due to land use and climate changes, investigations into soil erosion and sedimentation, evaluations of best management practices, and the assessment of risks [45,46]. Findings of the review suggested that the SWAT model is flexible and robust enough to model different Mediterranean watersheds, environmental, and hydrological processes [45]. Additionally, the examined applications of SWAT exemplify the model's versatility in accommodating diverse temporal and spatial scales [45]. In spite of what has been said, the primary obstacle highlighted in the report is the insufficient availability of essential data required for the configuration and calibration of SWAT modeling [45].

In a corresponding way, a review paper conducted in 2023 examined the application of the SWAT model in Karst Watersheds [47]. This special review provided a summary of 75 studies utilizing SWAT model in Karstic watersheds, with a specific focus on hydrological assessment within modified SWAT models [47]. The evidence of the survey highlights that both SWAT and SWAT + demonstrated noteworthy outcomes in modeling Karst watersheds [47]. However, it is worth noting that the research lacks comprehensive assessment of input data and their resolution and their influence on the output results [47]. In recent years, appreciable attention has been given to assessing the suitability of the SWAT model for application in coastal watersheds [47]. In the year 2022, a comprehensive review of 34 articles was conducted to dig into the feasibility of utilizing the SWAT model in these unique environments [48]. Unlike other watersheds, coastal watersheds present distinct attributes with the constant back and forth and flow of tides in coastal areas [48]. The findings of the study revealed a significant limitation of the SWAT model, namely its lack of ability to simulate the hydrodynamic flow of tides [48]. The study proposes several potential strategies to tackle this drawback, with the coupling of the SWAT model with a hydrodynamic model emerging as a particularly promising approach [48]. By unifying these models, researchers can enhance the accuracy and reliability of simulating tidal impacts in coastal watersheds [48]. Furthermore, the review highlighted another setback in the form of data input limitations, specifically related to the calibration of model for both flow and water quality owing to lack of available data points [48]. These findings accentuate the need for careful consideration of data constraints when utilizing the SWAT model in coastal watersheds, motivating further exploration and improvement in the modeling of hydrological processes in these dynamic environments [48].

Unlike previous reviews which often addressed broader applications of the SWAT model or focused on regional specifics, this review paper uniquely concentrates on the critical role of input data resolution in enhancing the accuracy of SWAT-based hydrological models. While past studies such as those documented in Refs. [40,41,49] provided comprehensive overviews of SWAT applications globally or specifically in regions like Africa, they did not systematically delve into how varying resolutions of DEM, soil data, LULC, and weather data impact model accuracy. Current study fills this crucial gap by not only highlighting the necessity for high-resolution data to improve outcomes specifically in sediment yield and streamflow accuracy but also by offering up-to-date guidance on selecting appropriate data resolutions to optimize model performance. This approach not only extends the existing body of knowledge but also serves as a critical tool for researchers and practitioners aiming to enhance the precision of hydrological modeling in various environmental conditions. For a more detailed comparison of how these themes have been explored across various studies refer to Table 1, which outlined the contributions and limitations of numerous reviewed articles in comparison to current study.

**Table 1:** The available review research contribution and the current research contribution.

References	Study Focus	Methodology	Time Period	Studies Region	Key Findings	Study Limitations	Remark
[40]	Evaluation of SWAT model applications	Review and synthesis of conference papers	Includes a range of studies up to 2014, selected non-systematically.	Global, with focus on North America and Europe	Presented key global application trends of SWAT, covering hydrologic foundations, sediment transport, nutrient and pesticide transport, and scenario analyses	Limited discussion on the model's limitations regarding the complexity of hydrological processes	Provides a comprehensive overview of SWAT applications across various environmental and hydrological scenarios
[41]	Impact of data scale on hydrological modeling outputs	Review of past studies and synthesis	Includes a range of studies up to 2017, selected non-systematically.	Various, referenced multiple global studies	Higher resolution data can more accurately describe watershed characteristics but increase computational demand. Important to select data resolutions that maintain model efficiency without compromising accuracy.	Does not specify the exact time frame of the studies reviewed, may lack recent developments.	Provides a comprehensive overview of how data resolution impacts hydrological modeling, useful for developing, applying, and improving watershed models.
[45]	Review of SWAT applications in Mediterranean watersheds	Review of literature	Systematically covers studies from 2002 to 2022.	Mediterranean watersheds	presented the application of SWAT model on hydrological processes, covering the quantity and quality assessment of water resources as well as the impacts of land use and climate changes of the hydrological and environmental processes.	Limited discussion on the impact of input data resolution of the SWAT model on the accuracy of the results.	Provides the ability of the SWAT model to simulate Mediterranean watersheds with high efficiency on temporal and spatial scales
[47]	Assessment the hydrological processes in modified SWAT model in Karst watersheds	Literature review	Systematically covers studies from 2000 to 2022.	Global	provides a summary of 75 studies utilizing SWAT model in Karstic watersheds across different regions around the globe. the results show the capability of SWAT model to simulate the hydrological processes in Karstic watersheds	Limited comprehensive assessment of input data and their resolution and their influence on the output results	Provides a review paper to simulate the karst watershed using SWAT model around the world
[48]	Application of SWAT model in coastal watersheds	Review of literature and recommendations	Includes a range of studies up to 2022, selected non-systematically	Global	The study suggested merging the SWAT model with a hydrodynamic model to address the shortcomings of the SWAT model and make it suitable simulate the hydrodynamic flow of tides	The research did not discuss the impact of input data resolution on the accuracy of the results of SWAT model	The findings of the study revealed the weakness of the SWAT model to simulate the hydrodynamic flow of tides
[49]	Review of SWAT applications in Africa	Review of literature	Systematically covers studies from 2005 to 2019.	Africa	Reviewed 206 studies across multiple aspects like water resources, erosion, land-use, climate change impacts, and model parameterization. Highlighted the challenges and benefits of using SWAT in various African contexts.	Did not provide detailed methodological differences or comparative effectiveness between different SWAT applications.	Provides comprehensive insights into the application of SWAT across various environmental and hydrological scenarios in Africa, underlining its versatility and adaptive use in modeling diverse conditions.
[14]	Overview of SWAT model applications	Literature review and synthesis	Includes a range of studies up to 2015, selected non-systematically.	Global	Highlights the versatility of SWAT across different regions and climatic conditions. Discusses applications in nutrients, sediments, BMPs, climate change, and land-use impacts.	Specific methodological details of individual studies not discussed in depth.	This paper provides a broad synthesis of SWAT applications, emphasizing its growing global usage and diversity of applications in water resources assessment.
[43]	Review of SWAT applications in various hydrological processes	Literature review	Includes a range of studies up to 2022, selected non-systematically.	Global	Reviewed SWAT model's applications and advancements, highlighting its utility in modeling complex hydrological processes and its adaptability in changing environments. Noted a significant increase in SWAT-related articles over the past three decades.	The review may not fully cover all geographic regions equally, potentially underrepresenting the application of SWAT in less studied areas.	Highlights both the strengths and weaknesses of SWAT, with a focus on its evolution and continuous development over the years.
[48]	Review of SWAT applications in coastal watersheds	Systematic literature review	Includes a range of studies up to 2022, selected non-systematically.	Global, focusing on coastal watersheds	Only 3 % of SWAT studies focus on coastal areas. Highlighted the need for combining SWAT with hydrodynamic models for better simulation of tidal influences in coastal watersheds.	Limited integration of SWAT with hydrodynamic models, which restricts the capability of SWAT in handling tidal influences.	First comprehensive synthesis of SWAT applications in coastal environments, emphasizing the necessity of enhancing model capabilities for coastal watershed management.
Our review paper	Resolution and accuracy of input data for SWAT-based hydrological models	Literature review	Up to date	Global	Emphasized the critical influence of DEM, soil data, LULC, and weather data resolution on SWAT model accuracy. Noted the need for higher resolution in data to improve SWAT model outcomes, particularly for sediment yield and streamflow accuracy.	–	Provides a structured analysis and guidance on selecting the appropriate input data resolutions to optimize SWAT model performance across various hydrological scenarios.

### 1.3 Current research review motivation

While the use of the SWAT for hydrological modeling is widespread, there is still a critical need to systematically evaluate how the resolution of various types of input data affects model outputs. Previous studies have often focused on individual aspects of model resolution, such as the impact of DEM or climate data resolution, but a holistic view that integrates multiple factors remains scarce. This gap in the literature suggests an opportunity for a more comprehensive approach that can provide deeper insights into the interdependencies of data resolutions and their cumulative effect on modeling accuracy. Current research aims to fill this void by conducting an extensive review of existing SWAT models, assessing the impact of spatial and temporal resolution on the precision of hydrological predictions. By exploring these relationships, this study seeks not only to refine current understanding but also to guide future model applications and data collection strategies. The motivation behind this review is to enhance the scientific community's ability to select optimal data resolutions that balance computational efficiency with the accuracy needed for effective watershed management. Through this work, we aim to foster methodological advancements and offer robust recommendations that can lead to more reliable and actionable insights in hydrological modeling.

### 1.4 The literature review procedure

**Fig. 1:**
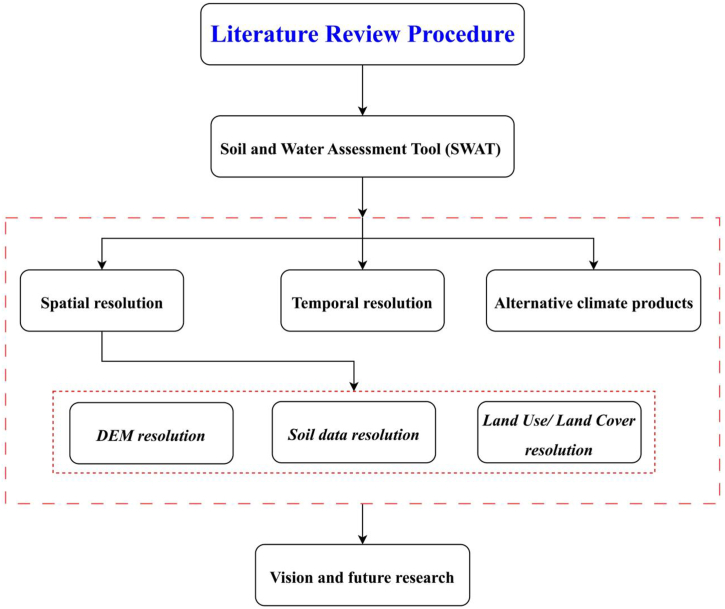
Methodology framework adopted for the literature review survey.

Current comprehensive literature review methodically explores the application of the SWAT by focusing initially on three primary dimensions: spatial resolution, temporal resolution, and the use of alternative climate products. Within these categories, special attention is given to the impact of spatial resolution on SWAT modeling, particularly analyzing the resolution of DEM, soil data, and LULC. Each aspect is scrutinized for its influence on the accuracy and effectiveness of the SWAT outcomes, highlighting the intricate interdependencies among them. This comprehensive analysis culminates in a discussion that lays out visions for future research, proposing directions that can potentially enhance SWAT's applicability and precision in hydrological modeling. For a visual representation of this systematic approach, please refer to the flowchart provided in Fig. 1.

### 1.5 Research objectives

The primary objectives of this comprehensive review are: (i) to provide an overview of both spatial and temporal resolution and to discuss the application of different climatic products within SWAT that represent suitable accuracy for streamflow and sediment yield for watersheds; (ii) to enable readers to more easily identify which spatial and temporal resolutions significantly impact the accuracy, efficiency of SWAT outputs, as well as which products are most suitable in the absence of or limited access to observed climate data, especially for transnational, limited, and ungagged basins; (iii) to examine ongoing challenges related to appropriate spatial and temporal resolution and alternative climate datasets in order to establish a framework for enhanced SWAT modeling; (iv) to review and evaluate the accuracy of SWAT input data derived from different data sources, as well as quantify the variation between these data sets over many temporal and spatial levels; (v) to provide results and recommendations from this evaluation that will be beneficial for SWAT and other hydrological model applications that can utilize the same input data as well as the same alternative climate data products.

## 2 Soil and water assessment tool (SWAT)

In the early 1990s, the interface between the Routing Outputs to the Outlet (ROTO) model and the Simulator for Water Resources in Rural Basins (SWRRB) model [50] was used to create the SWAT [51]. Since then, the program has undergone significant and continuous improvements since its first launch in 1994 [13,52], which was developed by the United States Department of Agriculture-Agricultural Research Service (USDA-ARS) [53,54]. SWAT has become a hydrological model that is broadly utilized across the world [15,55,56]; it is a physically based, semi-distributed, and continuous-time hydrological model for river basin simulation [13,15,40,[57], [58], [59], [60]]. SWAT is commonly used to simulate hydrological modeling processes [61], plant growth [62], erosion/sedimentation [63], nutrients [64], land management [65], climate change [66], land use changes [67], water resources management [68], stream routing [69], and pond/reservoir routing [70].

Generally, SWAT requires a DEM map, a soil map, a LULC map, and daily weather data for simulating the hydrological processes in river basins. An urgent need to enhance the model performance and ensure that the best observed streamflow is obtained [71]. Therefore, an evaluation of the temporal and spatial resolution of the input data sources must be conducted to present the best accuracy of simulating the streamflow and sediment yield in watersheds before applying them in SWAT. As previously mentioned, the higher the resolution of the input data, the more time, effort, and cost, as well as being less widely available for model users are required. Consequently, this study may provide insights into which data resolutions are most accessible and dependable for SWAT modeling. SWAT is utilized to simulate surface runoff, void zone processes (i.e., evaporation, infiltration, plant uptake, percolation, and lateral flows), and base flow [54]. For simulating surface runoff generation and soil infiltration, the Curve Number Method is used [72], while the Penman-Monteith Method is used to simulate evapotranspiration [73]. The soil moisture distribution in a profile is simulated using a bucket soil water model [74]; on the other hand, to predict lateral flow from soils to river networks, the kinematic storage model described by a research team was applied [75]. A recession constant for the base flow may be used to estimate the base flow [72].

In order to accurately evaluate SWAT results in light of observed data, statistical measurements and graphical comparisons are often used [57]. In the literature, numerous statistics have been reported for measuring the accuracy of SWAT estimations, such as Nash-Sutcliffe Efficiency (NSE), the root mean square error (RMSE), coefficient of determination (R^2^), Kling-Gupta Efficiency (KGE), and percent bias (PBIAS) [[76], [77], [78], [79], [80], [81]]. The NSE and R^2^ are the most frequently utilized statistics to assess the validity of SWAT model results [13,82,83]. Several studies recommended that NSE and R^2^ streamflow prediction results meet criteria of 0.5 and 0.6, with improved statistical criteria for evaluating simulations of streamflow that are deemed "good" or "very good" [78,79]. These studies were often used in the statistical analyses of the research evaluated here, where mentions of satisfactory or unsatisfactory results are made [78,79].

## 3 Spatial resolution

### 3.1 DEM resolution

DEM is a geospatial data product considered one of the crucial spatial input parameters in the hydrological modeling, which is a substantial form of satellite imagery or remote sensing data used in predicting a wide range of watershed variables [17,[84], [85], [86], [87]]. National mapping agencies create the majority of DEMs, for instance, the Ordnance Survey in the U.K. and the Instituto Geográfico Nacional in Spain [88]. Many sources of DEM data capture technologies that may have a considerable impact on DEM accuracy and quality, for instance, field survey [89], remote sensing [90], photogrammetry [91], LiDAR (Light Detection and Ranging) [92], etc. In current history, advancements have been achieved in the use of modern techniques like as LiDAR, which use direct distance measuring methodologies to calculate elevation, providing significantly higher degree of accuracy. Furthermore, global DEM products, which are known as GDEM, provide great ease and accuracy, such as the Shuttle Radar Topography Mission (SRTM), TanDEMx-TerraSAR-X add-ons for Digital Elevation Measurement, and the Advanced Spaceborne Thermal Emission and Reflection Radiometer (ASTER) [93].

DEM is a raster image that represents the physical characteristics of a watershed, such as the direction of flow, the drainage system, and slope [85,94,95]. The DEM is also used to obtain information about a channel's properties, including its width, depth, and slope [58,96]. The hydrological models' output is influenced by the DEM resolution [87,97], where watershed characteristics such as length, shape, slope, and surface area can be affected by the DEM resolution [[98], [99], [100]]. The DEM is used extensively in SWAT to simulate streamflow and sediment yield [58]. For instance, lower DEM resolution might result in lower slopes, which has a direct impact on the delineation of sub-basins and the formation of stream networks [101].

**Fig. 2:**
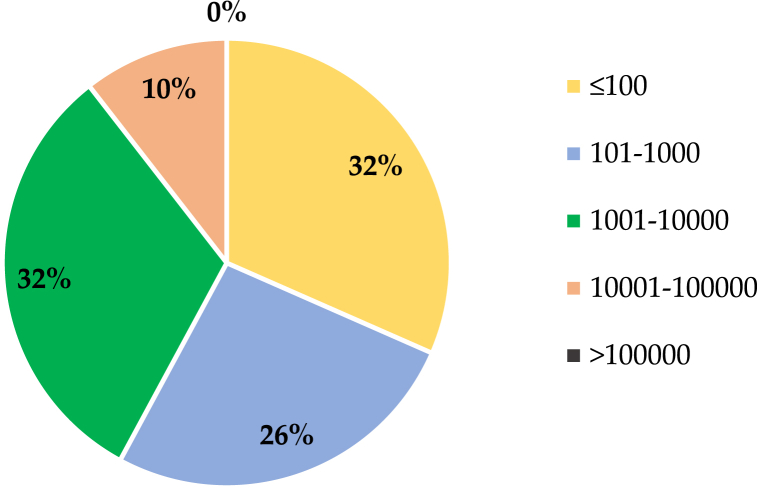
Distribution of catchment size ranges (in percentages) used in SWAT model studies applied DEM resolution. Each segment represents the proportion of studies that utilized specific catchment size categories, reflecting their frequency in hydrological modeling.

Numerous studies have been conducted to identify the best source and resolution of DEM. The studies have been conducted using data from SRTM, The Advanced Spaceborne Thermal Emission and Reflection Radiometer (ASTER), or LiDAR or data generated based on terrain measurements or topographic maps, which are available under the category of DEM resolution effects. All the studies have been published since the beginning of this century. As shown in Fig. 2, the selected studies were grouped based on catchment size. Fig. 2 shows that most of the studies were concerned with the study of small and medium watersheds, while very few (approximately 10 % of the total studies) were concerned with large catchments. According to Tan et al. (2021) [3], a basin is categorized as "large" if its size exceeds 100,000 km^2^, while it is considered "small" if the basin size is less than 100 km^2^. The percentages (%) in Fig. 2 illustrate the distribution of catchment size ranges used in SWAT model studies that applied DEM resolution in SWAT modelling. Each segment shows the proportion of studies within specific catchment size categories, reflecting how frequently each size range is utilized in hydrological modelling.

Numerous studies have shown that the hydrological model outputs are affected by DEM resolution [58,102,103]. A reserach study investigated the DEM resolution effect on surface runoff by using SWAT in a small watershed of 250 km^2^ [104]. The results demonstrated that the higher DEM resolution produced higher surface runoff. A number of investigations assessed the effects of DEM resolutions (30, 100, 150, 200, 300, 500, and 1000 m) on the performance of SWAT, identifying the uncertainties in predicting the surface runoff and sediment yield [17,18]. They found that stream networks, watershed delineation, and sub-basin categorization are all affected by DEM resolution. In addition, the coarse DEM resolution yields lower estimates of streamflow and sediment yield, while the finer resolution minimizes the uncertainties in the model estimations. Some researchers evaluated the impact of different DEM resolutions (30 m, 90 m, and 300 m) [105]. They found the streamflow predictions were far off when the models used DEM at 300 m resolution. The results also show that using resampled DEMs with a higher resolution may not help SWAT estimate streamflow more accurately. A research endeavor investigated the impact of DEM resolution on the results of surface runoff and sediment yield [106]. The results revealed that when DEM resolution increased from 10 m to 130 m, the runoff decreased slightly by 2.05 % while the sediment yield dropped by 41.72 %. Several academic investigations used various DEM resolutions to simulate the streamflow and the sediment yield [98,107,108]. The authors found that the finer resolution of the DEM yielded substantially more accurate slope data, which means that sediment production would be significantly more variable. Multiple scholarly analyses evaluated the impact of DEMs with different spatial resolutions on the results of streamflow and sediment yield [85,[109], [110], [111]]. The results demonstrated that when the DEM resolutions are less than 90 m, the streamflow calculations are not affected in SWAT. In contrast, the model is sensitive to DEM resolutions of less than 90 m in the case of sediment yield estimation. The DEM with a resolution of 30 m was found to be the most accurate for estimating the surface runoff and sediment yield as well as recommended that DEM (30 m) be used as an input for water resources instead of a finer resolution of DEM to save model calibration and validation time. Various scientific inquiries examined the performance of SWAT by using different DEM resolutions in small and large watersheds [58,99]. The studies demonstrated that when the DEM resolution is between 20 m and 60 m, the values of monthly streamflow and sediment yield will be better. A research effort also used small and large watersheds [103]. The authors advised using a finer resolution of 30 m or less in SWAT to accurately predict the hydrological characteristics of the watershed. Furthermore, multiple simulation studies examined the influence of DEM resolution (30 m–1000 m) [86,112,113]. Surface runoff was shown to be insensitive to variations in DEM resolution, in contrast to sediment output, which was found to be substantially impacted by changes in DEM resolution.

**Fig. 3:**
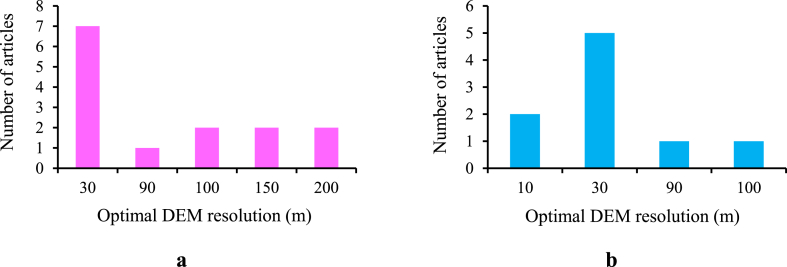
Comparison of optimum DEM resolution in the reviewed studies which using SWAT model (a) streamflow (b) sediment yield.

Based on the literature review, it has been found that the optimum DEM resolution that gives the best results for streamflow “Fig. 3a” and sediment yield “Fig. 3b” is 30 m, especially in the comparative studies that have been conducted in the last six years. Fig. 3 shows a clear preference for a resolution of 30 m in both the simulation of streamflow and sediment yield in SWAT. It is recommended that the coarser DEMs be utilized with care because the small watershed areas might be sensitive to DEM grid sizes. All studies proved the impact of the high DEM resolution on the sediment yield estimation, while many studies noted that the accuracy of surface runoff was not affected much by the increased DEM resolution, especially in flat watersheds. The surface runoff affected by the changes in DEM resolution in the case of mountainous watersheds. In mountainous watersheds, movements in watercourses or borders are often small and related to the shape's generalization within the pixel size range, so it requires a small pixel size in order to delineate the watersheds properly.

Ultimately, it can be concluded that emphasis should be given to study the DEM before modeling in order to establish which resolution in SWAT would work ideally for the watershed and thereby decrease model uncertainty. It is best to choose the DEM data resolution for the SWAT based on the desired watershed response, the process presentation, and the specific study area. However, the highly resolution data typically requires more cost, time, and computer memory.

### 3.2 Soil data resolution

Soil properties are required for hydrologic analysis since soil is the most significant hydrologic factors influencing watershed responses to rainfall events. Soil is made available for crop absorption and reduces surface evaporation while enhancing water efficiency and production [[114], [115], [116], [117]]. In hydrologic models, the physicochemical information of soil is one of the crucial inputs in the model that helps in the evaluation and analysis of the output. For instance, sandy soils generate less surface runoff and a high rate of infiltration is permissible, whereas clay soils produce more runoff and low rates of infiltration [118,119]. A great deal of work has gone into figuring out how to develop soil databases, which is a widely used approach in hydrologic modeling [[120], [121], [122], [123]]. Since the end of the 1980s, international organizations have been striving for the development of worldwide databases on soils to address this problem [124,125]. State Soil Geographic Database (STATSGO), The Soil Research Geographic Database (SSURGO), and Food and Agricultural Organization (FAO) are the most commonly used digital soil databases for the hydrological modeling [119]. For more than 30 years, the FAO/UNESCO World Soil Map was the only source of global soil information that was consistent. FAO maps take into consideration the dominant soil structure over the study area on a scale of 1: 1,000,000 [124].

In the United States, SSURGO and STATSGO are the most regularly used soil databases. The Natural Resources Conservation Agency (NRCS) created these digitized soil datasets, which are utilized as input data in water quality simulation models. SSURGO-level maps are inaccessible in every location, so STATSGO maps are generated using topography, vegetation, climate, data on geology, and Landsat images. STATSGO uses a mapping scale of 1:250,000 [126]. The base map was constructed using 1:250,000 topographic quadrangles from the US Geological Survey. Each quadrangle map has 100 to 400 soil polygons. SSURGO is a highly accurate soil dataset, with maps created at scales ranging from 1: 15,840 to 1: 31,680 [127].

**Fig. 4:**
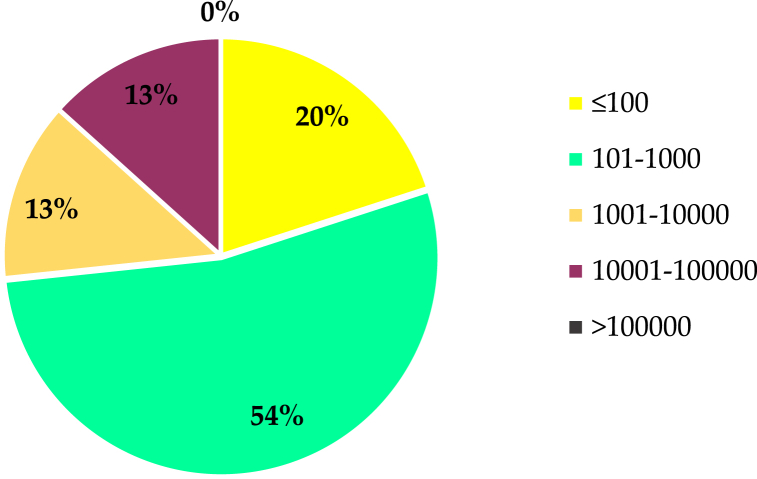
Distribution of catchment size ranges (in percentages) used in SWAT model studies applied soil data resolution. Each segment represents the proportion of studies that utilized specific catchment size categories, reflecting their frequency in hydrological modeling.

Very limited studies have been performed with SWAT to investigate how soil characteristics affect hydrologic processes. Overall, only 15 articles were included in this review, which is available under the category of Soil Data Resolution Effects. The selected studies were classified depending on catchment size, as shown in Fig. 4. The figure shows that most of the studies were done in what are called "small" catchments, which are smaller than 1000 km^2^. Only 14 % of the studies were performed in catchments larger than 10,000 km^2^, which emphasizes that the comparison between different soil databases in large catchments is still limited. The percentages (%) in the figure illustrate the distribution of catchment size ranges used in SWAT model studies that applied soil data resolution in SWAT modelling. Each segment shows the proportion of studies within specific catchment size categories, reflecting how frequently each size range is utilized in hydrological modelling.

Some of these studies examined how different resolutions of soil data affected streamflow and sediment yield, focusing on a comparison between using the low-resolution STATSGO database and the high-resolution SSURGO database. In the Thyle Catchment, Belgium, a research effort investigated the effect of two soil data resolution on SWAT simulation (1:500,000 and 1:25,000) [128]. The authors indicated SWAT was shown to be highly dependent on soil data resolution. Various exploratory case studies compared the performances of STATSGO and SSURGO for simulating the streamflow and sediment yield in different watersheds in the USA [[129], [130], [131], [132]]. The results showed that the SSURGO database outperformed STATSGO in terms of streamflow and sediment yield estimation because SSURGO has more detail than STATSGO. While multiple examinations found that the spatial resolution of the two soil datasets had no effect on estimating streamflow and sediment before and after calibration [133,134].

Several studies have examined the effect of soil data accuracy using other soil databases. A scholarly analysis examined the performance of the Soil Land Inference Model (SoLIM) approach compared with a 1:1,000,000 soil map [123]. SWAT successfully simulated streamflow in both soil databases, whereas the model that used SoLIM as a soil database performed better in the simulation of peak flow events. A scientific research used two different soil databases, FAO and the Genetic Soil Classification of China (GSCC) to recognize the impact of soil data resolution on streamflow and sediment production [135]. The findings revealed that the FAO database performed better than other database in modeling streamflow before calibration. This indicates that the both databases have good performance in streamflow simulation, while GSCC has a better possibility of estimating sediment yield. In Ethiopia, an examination examined the influence of soil characteristics on two watersheds (Gumara and Ribb) [117]. These watersheds were similar in size, topography, and land cover, with a slight difference in precipitation and evaporation, while the soil was significantly different. The results indicated that the difference in soil characteristics has a considerable impact on the hydrological responses of the watersheds. It has been recommended that the necessity to develop fine-resolution soil databases and focus on soil characteristics in watershed analysis. An academic inquiry studied Harmonized World Soil Database (HWSD) and TAMED-SOLnto show the extent of their impact on SWAT simulation in Morocco [136]. The HWSD is a global low-resolution soil database, developed by the FAO, the Institute of Soil Science, the International Institute for Applied Systems Analysis (IIASA), and the Chinese Academy of Science [124]. TAMED-SOIL is the result of an 18-month investigation by the research team of investigator with a scale of 1:50,000 created by DMN-Morocco (National Direction of Meteorology), INRA-Morocco (National Institute for Agronomic Research), IDRC-CANADA, and ICARDA-SYRIE [136]. An investigative study concluded that soil data quality and resolution affect all hydrological cycle components [136]. An empirical investigation examined the impact of the Africa Soil Information Service (AfSIS 250 m) database on increasing the flow prediction accuracy by comparing it with four other soil databases [137]: the HWSD, HWSD with data gaps filled from FAO's DSMW (hereafter referred to as HWSD3), Digital Soil Map of the World (DSMW) and Abay (Upper Blue Nile) basin master plan study soil map in Upper Blue Nile, Ethiopia. The AfSIS data contains information about a grid of soil characteristics such as sand, silt, and clay for as much as six layers of soil depth [138]. The Africa Soil Profiles database and the AfSIS Sentinel Site database have been used to compile the AfSIS soil database covering the years 1950–2012 with a spatial resolution of up to 250 m and 1 km to generate detailed soil properties [139]. A research project, found that even with the most detailed AfSIS soil, streamflow simulations were only marginally improved [137]. An academic inquiry investigated the impact of specified soil database (SSM) and FAO database resolutions on the performance of SWAT for simulating the surface runoff in Turkey [140]. The results showed that the SSM increased the accuracy of runoff better than FAO soil database in small watersheds.

Progress in understanding the influence of soil data resolution on the hydrological response of watersheds requires a thorough assessment of the previous studies, especially the effect of watershed areas. A scientific research analyzed a large humid watershed with an area of 15,535 km^2^ in the Xinjiang River basin, China, to evaluate the effect of soil data resolution on streamflow [141]. The analysis showed that improving the resolution of soil data does not necessarily result in more accurate surface runoff estimations in large-scale watersheds. Whereas, in a small watershed (Olešná reservoir, the Czech Republic, with 33 km^2^), a exploration compared the performance of a global dataset, Soil Grid, and a dataset derived from local historical data, which has more details for prediction of streamflow using SWAT [142]. This research found soil legacy data performed better at predicting streamflow [142].

Only a few studies have been evaluated the effect of soil data resolution on SWAT output. Most of these studies were simulated in watersheds with an area of less than 1000 km^2^, while studies in large watersheds are still limited. Most of the studies in the USA focused on the comparison between two types of soil databases, which are the high-resolution SSURGO and the low-resolution STATSGO. The results demonstrated that STATSGO slightly performed better than SSURGO for simulating the streamflow and sediment yield before calibration, while SSURGO performed better after the calibration. Overall, the majority of the studies estimated that improving the resolution of soil data does not necessarily result in more accurate streamflow and sediment yield predictions. On the other hand, producing specialized soil maps using a limited number of soil samples or refining existing soil maps may significantly improve the accuracy of SWAT for estimating the sediment yield and surface runoff. The more detailed soil data requires more time, effort, and processing power to set up and calibrate a model, especially in large watersheds. So, before deciding on the amount of soil data resolution to use, modelers should think about the benefits of using this resolution. Hence, more investigation on the impact of soil database resolution on SWAT outputs is required, especially in large catchment areas in which the nature of the soil changes from strong rocks to weak soils, to recognize specifically the impact of soil data resolution on the validity of streamflow and sediment production.

### 3.3 Land use/Land cover resolution

LULC is an important factors affecting the watershed hydrology because it has significant effect on hydrological processes [[143], [144], [145], [146], [147]]. Models of watershed hydrologic processes may be influenced by the LULC data, which alters the rates of interception, evapotranspiration, infiltration, and groundwater recharge [145,[148], [149], [150]]. In hydrological modelling, spatial resolutions, classifications, and different interpretation accuracies of LULC datasets can result in different resolutions of LULC datasets [145,151,152]. It is critical to have a comprehensive realization of the impact of using satellite data with varying spatial resolutions on the response of hydrological models. In general, the more accurate the input data is, the more accurate and reliable the modeling results will be [153,154], Nonetheless, this is not always valid.

**Fig. 5:**
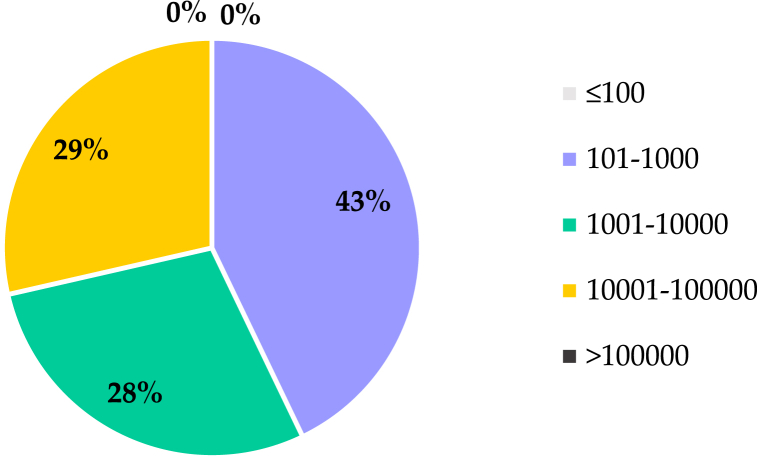
Distribution of catchment size ranges (in percentages) used in SWAT model studies applied LULC resolution. Each segment represents the proportion of studies that utilized specific catchment size categories, reflecting their frequency in hydrological modeling.

The paucity of studies that dealt with the effect of the spatial resolution of LULC in SWAT model. The reviewed studies were categorized according to the size of the catchment area, as shown in Fig. 5. The percentages (%) in the figure illustrate the distribution of catchment size ranges used in SWAT model studies that applied LULC resolution in SWAT modelling. Each segment shows the proportion of studies within specific catchment size categories, reflecting how frequently each size range is utilized in hydrological modelling. This categorization shows that all studies are considered medium-sized. However, no study has taken into consideration the effect of the spatial resolution of LULC on the accuracy of SWAT outputs in the catchments with areas of less than 100 km^2^ or greater than 100,000 km^2^.

Several conclusions have been obtained about the influence of LULC data resolution on hydrological modeling. A theoretical analysis examined the effect of high-resolution LULC data classified with the object-oriented image analysis (OOIA) technique and low-resolution LULC data classified with the pixel-based maximum-likelihood (PBML) technique on the accuracy and reliability of streamflow simulation [151]. According to the findings, the low-resolution model had somewhat greater prediction reliability. An investigation used a spatial resolution of 1 m from Digital Globe and 30 m from Landsat to examine the effect of spatial resolution on total suspended solids (TSS) load [155]. The results demonstrated that TSS load was better predicted by models employing 1 m datasets for the study area. However, the 1 m data is significantly more expensive when compared with the 30 m data. Various grounded theory projects used different resolutions of LULC [152,154,156,157]. The results showed that the fine-resolution LULC datasets could not guarantee an accurate streamflow simulation or give better statistical findings; they also had no significant influence on the model's calibration. The results indicated that low-resolution LULC data is often adequate for obtaining satisfactory results while lowering processing and calibration efforts, hence saving time and money. Some studies took into consideration the complementary and interactive impacts of the DEM and LULC resolutions on surface runoff estimation. Several studies showed the runoff values were more sensitive to changes in LULC resolution in comparison with changes in DEM resolution [158,159]. This is due to the fact that CN is estimated based on land use, mean slope, and soil types within a basin.

The effect of LULC resolution on SWAT output has only been investigated in limited studies despite its crucial importance. All studies used medium watershed areas, while no study showed the effect of spatial resolution of LULC on the accuracy and validity of SWAT outputs in small and large watersheds. It is clear that there are shortcomings in the studies that dealt with the impact of LULC resolution on the accuracy of sediment yield. LULC increases soil cohesion and thus reduces erosion. Therefore, this aspect should be given a lot of attention in future studies. Accordingly, the fine resolution of LULC datasets may not always provide accurate streamflow simulations. In contrast, the fine resolution of LULC provides an accurate simulation of sediment yield, but this costs significantly more. The studies also showed that the input data for the specific watersheds may not always provide the same results in different watersheds; therefore, it may not be suitable for other watersheds. Advancing in understanding the effect of LULC resolution on the results of watershed modeling needs a strong emphasis and more studies with small and large watersheds, but there should be attention that high resolutions need more effort, time, and cost. Nevertheless, the choice is dependent on the objective of the research and the anticipated results.

## 4 Temporal resolution

Rainfall is a crucial factors impacting the hydrological processes of river basins [22,[160], [161], [162], [163]]. The primary source of uncertainty in SWAT modeling and flood forecasting for catchments is the temporal resolution of rainfall [[20], [21], [22]]. The temporal resolution of precipitation may considerably impact the streamflow in hydrological modeling [22,54]. Higher temporal resolutions of precipitation may enhance the streamflow in the hydrologic models, particularly at the peak of a flood wave [22,54,164]. SWAT is used in hydrological modeling to compare two different rainfall-runoff models: the empirically based Curve Number (CN) method [165], or the physically based Green-Ampt method [166]. The CN method, which is used for daily rainfall models, is a conceptually simple model with empirically based relationships between daily rainfall, soil type, and land use, without taking the duration of storm events or rainfall intensity into consideration. On the other hand, the Green-Ampt method is used for sub-daily rainfall models with a time step of 1 min, which is based on physical relationships that consider rainfall duration and intensity of storm events [163,[167], [168], [169], [170], [171]]. Most studies use the CN method. The reason for this may be the lack of sub-daily rainfall data, particularly for large watersheds and developing countries [172]. However, if sub-daily rainfall data is obtainable, its application is promising to more accurately simulate streamflow and other hydrological processes in light of the more accurate assessment of rainfall duration and intensity [163].

**Fig. 6:**
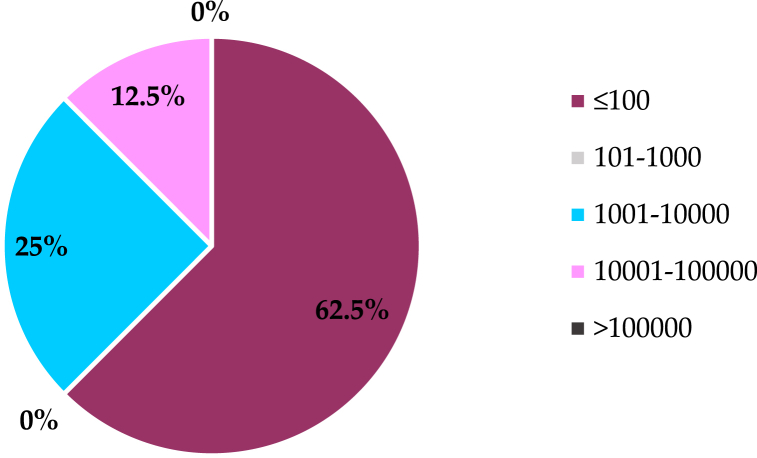
Distribution of catchment size ranges (in percentages) used in SWAT model studies applied temporal resolution of rainfall. Each segment represents the proportion of studies that utilized specific catchment size categories, reflecting their frequency in hydrological modeling.

Due to the limited studies, it is difficult to compare the results of the sub-daily Green-Ampt and daily CN methods in SWAT. The reviewed studies were categorized according to the size of the watersheds. Most of the watersheds were small, and only 12 % were large in size. However, no study compared the results of daily and sub-daily precipitation in watersheds with a size greater than 100,000 km^2^, as shown in Fig. 6. The percentages (%) in the figure illustrate the distribution of catchment size ranges used in SWAT model studies that applied temporal resolution of rainfall in SWAT modelling. Each segment shows the proportion of studies within specific catchment size categories, reflecting how frequently each size range is utilized in hydrological modelling.

The studies evaluated the impacts of using the daily CN method and the sub-daily green-Ampt method on streamflow and sediment yield by using SWAT. A scientific research evaluated the use of the CN method and the Green-Ampt method by applying 1-h rainfall records in the Goodwin Creek Watershed, north central Mississippi [167]. The results found that the Green-Ampt method was the most efficient method for modeling daily streamflow data. While A number of investigations studied the impact of the CN method and Green-Ampt method on predicting daily streamflow on a small catchment in the Unilever Colworth estate, England, and a large catchment in the San Joaquin River watershed, USA, respectively [168,169]. These studies indicated that the CN method was more accurate in predicting daily streamflow. In multiple scholarly inquiries, the effect of rainfall data accuracy on the daily streamflow in small watersheds was studied [163,170,173]. An examination studied the Lost Creek Golf Course Area (LGA) watershed in Texas, USA, with an area of 1.9 km^2^ [173]. A research studied the Jawoon-ri watershed in Korea (0.8 km^2^) [170]. While another research endeavor studied a catchment (1.81 km^2^) in northeastern Germany [163]. The results found that the hourly precipitation time steps with the Green-Ampt infiltration method were much better at predicting runoff and had higher accuracy compared with the daily rainfall data. Applying time steps (less than 1 h) has increased the surface runoff in the case of soils that are less permeable, such as loamy or clayey soils, as well as in the case of mountainous or urbanized catchments. Sub-daily rainfall time steps have no influence on daily streamflow for catchments with soils of low runoff potential in lowland areas. Ultimately, the authors recommended using the smaller precipitation time steps in the case of sediment or solute transport modeling. Various academic examinations investigated the effect of temporal resolution on the daily streamflow in river basins of medium size [162,171]. A scientific study examined the effect of high rainfall resolution on SWAT performance in the Upper Huai River Basin, which has a total surface area of 5803 km^2^ [162], while another exploratory factor analysis studied the Xiaohong River Basin, China, which has a total surface area of 4417 km^2^ [171]. The results showed that SWAT is more sensitive to the hourly rainfall model compared to the daily rainfall model, especially during flood season.

Rare studies have examined the effect of temporal rainfall resolution on the output of SWAT. In developing countries, the scarce of high-resolution rainfall statistics is a major impediment to applying the sub-daily routine in SWAT. Most of these studies were simulated in watersheds with an area of less than 100 km^2^, while few studies showed the influence of temporal precipitation resolution in vast river basins. Finally, the use of sub-daily precipitation data with high temporal resolution may be promising for predicting streamflow more precisely, especially on days with heavy rainfall and longer rainfall hours, which take into consideration the duration and intensity of rainfall with more precision. It is also worth noting that the smaller precipitation time steps can be used in the case of sediment or solute transport modeling. It is worthwhile to keep in mind that using finer temporal resolution can increase the computational cost. Therefore, it is important to achieve a reasonable compromise between model accuracy and efficiency. The impact of temporal rainfall resolution on the accuracy of SWAT outputs should be investigated more extensively than what is currently available, especially during flood season.

## 5 Alternative climate products

One of the difficulties encountered by hydrological modelers in developing countries is the lack of meteorological data [[174], [175], [176]]. This is due to the short periods of weather observations, lack of weather stations, and weather records that contain gaps or missing data, increasing the difficulties of model predictions based on such data, particularly in large catchments [[176], [177], [178]]. Precipitation is a crucial weather variable that must be included in hydrological models. Hence, proper representation of precipitation variability is critical to accurate simulation and estimation in hydrological modeling [[179], [180], [181], [182]]. Alternative climate data sources, including satellite precipitation, gauge-based gridded data, reanalysis products, and ground-based weather radar, were utilized to solve the challenge of rain gauge observations of limited or ungauged river systems [3]. In recent decades, satellite technology has become an indispensable instrument and has seen great progress. A number of satellite precipitation products were released for climate data retrieval and monitoring because of their capacity to cover almost all of the planet's surface. Satellite products include spatial resolutions ranging from 0.1° to 0.25° and temporal resolutions ranging from 30 min to daily scales. For example, the 1997-released Tropical Rainfall Measuring Mission (TRMM) Microwave Imager (TMI) continually records tropical and subtropical precipitation every 3 h [183]. In addition, the interpolation of hundreds of regional and global rain gauges was used to generate gridded climate data sets. Data from over 100,000 climate stations across the globe is compiled every day by the Global Historical Climate Network Daily (GHCND), including daily rainfall, daily minimum and maximum temperatures, snowfall, and snow depth [184]. When it comes to using reanalysis climate products, among the most extensively used datasets are those produced by the National Centers for Environmental Prediction (NCEP), Climate Forecast System Reanalysis (CFSR), and the North American Regional Reanalysis (NARR) [185]. Both of which take into account physical and dynamic processes and irregular observations and models [25].

**Fig. 7:**
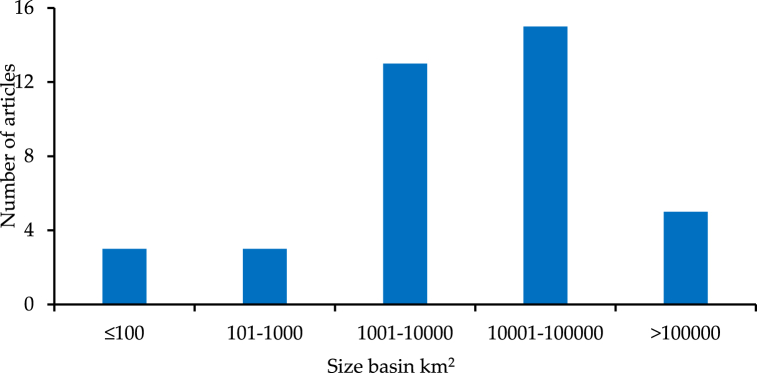
Comparison of the basins size of the reviewed studies that applied alternative climate sources in SWAT-Based models.

SWAT was commonly used worldwide to simulate precipitation observations. In total, more than 150 articles are available under the "climate data effects" category, most of which were published in the past six years. The widespread use of SWAT around the world in recent years is possibly the reason for the increment [81]. In this study, the focus was on recent studies; a total of 30 studies were chosen. After that, the chosen studies were grouped based on basin area, as shown in Fig. 7. The figure demonstrates that the river basins' area for the majority of the studies is concentrated in the "middle" and "large" river basins. The proportion of medium basins (with an area ranging between 1001 and 10000 km^2^) was 31.4 %, and the proportion of large basins (with an area ranging between 10001 and 100000 km^2^) was 37.1 %. The areas of small basins (with an area of less than 100 km^2^) were represented by limited studies, the percentage of which did not exceed 8.6 %.

**Fig. 8:**
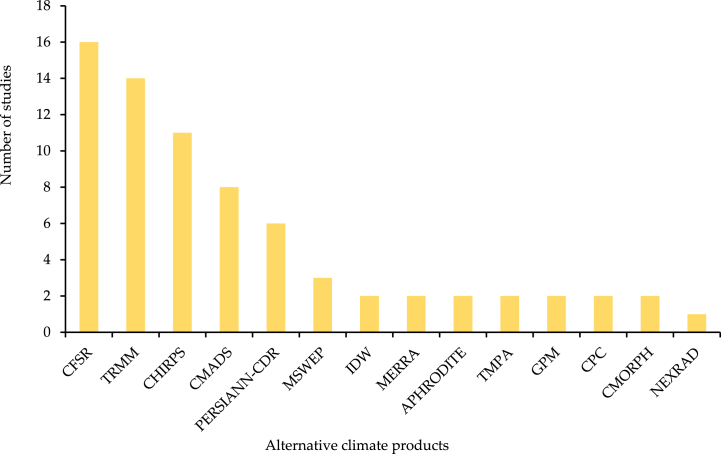
Number of studies in current study that use of alternative climate products in SWAT modeling.

Since 2004, SWAT modeling has used 40 alternative climate products [3]. Precipitation data is only provided on a daily basis by the vast majority of these products. In the current study, SWAT modeling relies heavily on CFSR and TRMM data, two of the most extensively used alternative climate datasets, followed by CHIRPS and PERSIANN-CDR. Furthermore, IDW is primarily an interpolation method, not a direct climate product or dataset. It was included in the study to illustrate the breadth of methods and data sources commonly used in SWAT modelling for input data preparation, particularly where raw data might be sparse or irregularly distributed. This interpolation method is often utilized to create gridded datasets from point measurements, which can then be used as input for SWAT as shown in Fig. 8.

A scientific research were one of the first to implement an alternative climate product in SWAT [186]. It was used with National Weather Service (NWS) high-resolution radar rainfall data, known as the Next Generation Weather Radar (NEXRAD). The results revealed that NEXRAD has good performance in simulating streamflow and could be used instead of data from weather stations.

The widespread use of the CFSR may be related to the ease with which SWAT-formatted data can be obtained, as well as the fact that CFSR data does not need to be processed before being entered into SWAT. In addition, CFSR records rainfall, maximum and minimum temperature, relative humidity, wind speed, and solar radiation over a long-time frame, from 1979 to 2014. Many studies dealt with the performance of CFSR in comparison with other products and in various watersheds around the world to identify the extent of its impact on streamflow simulation and the possibility of using it as an alternative climate product. Several scholarly explorations have performed SWAT simulations using only CFSR data and compared the results with conventional weather data [176,187]. An academic investigation studied the simulation of streamflow and soil loss in three watersheds in the Blue Nile Basin, Ethiopia [187]. The results demonstrated that conventional weather data led to very good performance for estimating streamflow and soil loss, while CFSR datasets led to unsatisfactory results. Furthermore, conventional weather data remained the most reliable and accurate source of data. In the event that inadequate gauge data, a scholarly analysis suggested that CFSR might be used as an alternative data input [176]. For the upper Gilgel Abay basin in Ethiopia, A scientific inquiry examined the performance of the CFSR, TRMM, and CHIRPS and gauged data to simulate monthly and daily streamflow [182]. The findings indicated that gauged data outperformed all three alternative climate products in streamflow simulation. On the other hand, the CFSR precipitation datasets had unsatisfactory results in daily and monthly streamflow simulations compared to the other products used in this study. This is consistent with what found in simulating the streamflow in the Lake Ziway Basin, Ethiopia [188], and found in simulating the streamflow and sediment in the Lancang River Basin (ULRB), China [189]. The results also showed that the CFSR dataset is not appropriate for any hydrological modeling. Diverse scientific studies investigated the performance of CFSR and CHIRPS products against the observed streamflow gauging stations [190,191]. Two watersheds have been simulated by a simulation study for each of the following countries: the USA, Spain, Brazil, India, and Ethiopia [190]. While the other case study was in the Baro-Akob River Basin, Ethiopia [191]. The results in both studies demonstrated that the CHIRPS gave a better performance for the prediction of streamflow than the CFSR. The CFSD was also not the best performing in the studies conducted by a research project in the Grande de San Miguel (GSM) River Basin, El Salvador [192], and an academic exploration, in the Chenab River Basin, India [193]. Finally, a variety of scholarly investigations reached the same conclusion [190,192,[194], [195], [196], [197]].

TRMM was a collaborative satellite mission between NASA and the Japan Aerospace Exploration Agency (JAXA) over a time frame from 1997 to 2015 that spanned 50°N–50°S with 3-h temporal resolution and 0.25° spatial resolution [183]. Several studies have examined TRMM to verify its accuracy and reliability in streamflow simulation using SWAT. A scientific research studied the performance of TRMM rainfall product to compute the streamflow by comparing it with the streamflow from observed rainfall data in the Tiaoxi watershed, Taihu Lake, China [198]. According to the findings, the monthly streamflow simulation was accurately predicted by TRMM rainfall product. Moreover, a research effort found TRMM produced better performance than CHIRPS when they were utilized to simulate the monthly and daily streamflow in the Lower Lancang-Mekong River Basin, China, with gauge observation data and inverse distance weighted (IDW) products [199]. As well, the results of different research efforts showed that the simulation of the monthly streamflow was accurately predicted by TRMM rainfall product in the Mekong River Basin (MRB) in Southeast Asia and in the Xihe River basin, China, respectively [195,200]. An empirical investigation, utilized TRMM, CFSR, the Asian Precipitation Highly Resolved Observational Data Integration towards Evaluation of Water Resources (APHRODITE), and the Global Precipitation Climatology Center (GPCP) to assess the performance of SWAT of simulating the streamflow [194]. The results showed that TRMM, APHRODITE, and GPCP are more convenient for simulating hydrological models in India. However, TRMM performed poorly when compared to CHIRPS data, where the latter achieved better results in streamflow simulation. With more than three decades of data, CHIRPS has been able to create a time series of gridded rainfall by combining rain gauge station data and satellite data with 0.05^0^ spatial resolution. The dataset includes climatology for monthly rainfall, rainfall observations from global or regional meteorological sources, Climate Hazards Group Rainfall Climatology (CHP Clim), TRMM 3B42 rainfall product, geostationary thermal infrared satellite observations, and atmospheric model precipitation fields from the NOAA Climate Forecast System [201]. A theoretical analysis studied the performance of CHIRPS with three precipitation datasets, incorporating Observed Precipitation (OP), TRMM, and Inverse Distance Weighting data (IDW) in the Adige river basin, Italy [180]. The results showed that CHIRPS had a better performance in estimating streamflow and can be used to estimate the data-sparse in the Alpine region. While TRMM dataset leads to inadequate results in streamflow simulation when used in the Alpine region. Additionally, according to an observational study, the CHIRPS showed the best performance when compared to TRMM and CFSR, especially when using the high spatial resolution of CHIRPS (0.05⁰) for simulating monthly and daily streamflow in the upper Gilgel Abay Basin, Ethiopia [182]. Furthermore, a case study found CHIRPS surpassed TRMM product in the Lake Ziway basin, Ethiopia [188]. A research endeavor observed that the two products performed better when they assessed the performance of sixteen rainfall products in simulating the streamflow in the Tungabhadra river basin, India [202].

A scientific study compared TRMM against a gauge-based Indian Meteorological Department (IMD) to assess the effectiveness of SWAT to simulate monthly and daily streamflow and suspended sediment load in the Marol watershed, Krishna River basin, India [203]. The results demonstrated that IMD was performing better than TRMM. However, high sediment load and flood events were usually underestimated in both datasets. In addition, An academic investigation utilized TRMM against global precipitation mission (GPM) IMERG-F v6 to assess the daily and monthly streamflow pattern in Chenab River, Pakistan [204]. The results showed that the GPM is superior to TRMM. A scholarly analysis, also found that TRMM performance was poor compared to the Modern-Era Retrospective Analysis for Research and Applications (MERRA-2) and CFSR in simulating the streamflow in SWAT model Manafwa catchment, Uganda [205]. A research project have found the MERRA-2 precipitation dataset performed better [205].

In China, the China Meteorological Assimilation Driving Dataset for SWAT (CMADS) was created to provide high-quality meteorological data for East Asian meteorological and hydrologic applications [206]. CMADS is a new reanalysis climate data for SWAT modeling that contains climate variables such as rainfall, maximum and minimum temperature on a daily scale, average temperature, specific humidity, relative humidity, wind speed, solar radiation, soil moisture, average atmospheric pressure, and soil temperature. CMADS was converted to SWAT format, similar to NCEP-CFSR, so that modelers could easily access the data. Hydrological applications in China have made extensive use of the CMADS capabilities [197,207,208]. However, in other East Asian countries, such studies are still scarce. River basins in China have a relatively high number of studies looking at the effectiveness of CMADS for SWAT modeling. A study in the Xiang River basin, China [209], and another one in the Qujiang River Basin, China [210], studied the performance of CMADS. The findings demonstrated that the CMADS product offered the best performance in monthly and daily streamflow simulations. An investigation, in the Fuhe River Basin (FRB), China, evaluated the performance of CMADS by comparison with the CFSR product to simulate the streamflow [211]. The results showed that two precipitation datasets yield good streamflow simulation results, with some advantage to the CMADS, which was able to predict the extreme streamflow events slightly better than the CFSR product. A scholarly analysis in the Jing River and Bo River Basins [212], and second one in the Danjiang River Basin (DRB) [213], demonstrated that CMADS has good representativeness and reliability in streamflow simulation. An evaluation of SWAT-based CMADS outside of China was carried out by a research effort in the Muda River Basin (MRB) [196], Malaysia, and Further evidence in the Cau River basin (CRB), northern Vietnam [214]. The results revealed that CMADS outperformed NCEP-CFSR in a monthly streamflow simulation.

In Peru and Ecuador, a new hydrologically corrected daily rainfall dataset at 0.18 spatial resolution over a time frame from 1981 to 2015 covering Peru and Ecuador has been developed by Ref. [215]. It is called RAIN4PE (Rain for Peru and Ecuador). A comparison between the new approach dataset and gauged rainfall datasets (CHIRPS, MSWEP, and PISCO) was achieved to assess the performance of RIAN4PE in estimating the streamflow. RIAN4PE was shown to be the most effective tool for streamflow simulation. In the Tigris River Basin (TRB), of which Iraq accounts for 56.1 %, Turkey for 24.5 %, Iran accounts for 19.5 %, and Syria accounts for 0.4 %, A predictive modeling inquiry evaluated the performance of four widely used satellites and gauged-based precipitation products to simulate streamflow, which are (PERSIANN-CDR), (APHRODITE), National Oceanic and Atmospheric Administration (NOAA) Climate Prediction Center (CPC), and Multisource Weighted-Ensemble Precipitation (MSWEP) data [216]. This study discovered that APHRODITE outperformed the other datasets in simulating the streamflow, whereas CPC performed poorly compared to the rest [216].

The contribution of climate products has increased in the last six years in watershed modeling due to limited recorded rainfall data or an inadequate rain gauge network. The climate products encompass four main categories: satellite precipitation products; gauge-based gridded data; climate reanalysis datasets; and ground-based weather radar. CFSR and TRMM are the most commonly utilized among the other climate products evaluated by SWAT modelers, followed by CHIRPS, CMADS, and PERSIANN-CDR (Fig. 8). In the majority of the studies, CHIRPS consistently outperformed the other alternative products in estimating the streamflow in watersheds with limited gauged data. The finer spatial resolution of 0.05^0^ may be responsible for the substantially improved performance of the CHIRPS dataset, which increases the dataset's effectiveness in capturing spatial variation in precipitation. On the other hand, CFSR performed poorly when compared to other products that were evaluated in several studies where it tended to overestimate streamflow in SWAT simulation. Although CMADS is one of the most trustworthy alternative climate sources in China (as well as in Vietnam and Malaysia), the open-source version is only accessible for 11 years for a period from 1998 to 2008), which limits its use in hydrological modeling. In Ethiopia, most studies showed that CHIRPS was a better choice for watershed simulations. Additionally, future research may provide additional testing of CHIRPS data in more diverse regions. Ultimately, the results showed that gauged data had a better performance in simulating streamflow compared with all other climate products. Selecting alternative climate products has a significant impact on model performance, model uncertainty, and parameter uncertainty in streamflow simulations. Employing various datasets for model simulations in a catchment can lead to different results. The uncertainties associated with alternative climate products result in unreliable measurement of basin hydrological response owing to overestimation or underestimate of streamflow simulation. In addition, this uncertainty cascade propagates to processes like erosion and pollutant transport, presumably resulting in various water management techniques or policies.

Given the necessity to identify the performance and anticipate the launch of new future climate sources, SWAT modeling is projected to continue comparing various alternative climate products to infinity. However, guidelines should be created for future SWAT studies to increase the consistency among studies that employ generally accepted products and statistical evaluation methodologies that account for both rainfall and temperature data.

## 6 Vision and future research direction

**Fig. 9:**
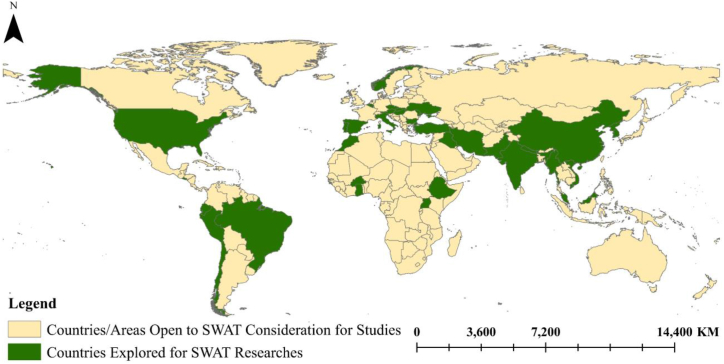
Geographical Distribution of Countries where SWAT Studies were Applied: Insights from this Literature Review. The map illustrates two categories: (1) countries that have been extensively explored for SWAT-related research (shown in dark green) and (2) countries/areas where SWAT is considered applicable for studies but may not yet have extensive research (shown in light green). (For interpretation of the references to colour in this figure legend, the reader is referred to the Web version of this article.)

The collective results presented in this comprehensive review offer compelling proof of the efficacy of SWAT as a valuable tool for addressing diverse water resource and land management challenges. The continuous backing of SWAT by both governmental and private educational organizations, coupled with its adaptable nature, has led to a surge in inventive applications and customizations, contributing to its widespread acceptance and implementation across the globe (Fig. 9). For future consideration, the literature review identified several areas that warrant further exploration and research in the context of SWAT's potential advancements. However, certain shortcomings identified in a subset of the studies presented in this section highlight the necessity for broader testing and targeted enhancements in the future researches. A number of studies in this analysis focus on calibrations related to the Runoff Curve Number (RCN), a parameter commonly examined in SWAT studies. These findings underscore the need for more research on RCN, including further exploration of the Green-Ampt method, which has received limited attention in SWAT studies. Additionally, alternative approaches to RCN in modified SWAT applications warrant further investigation [40].

Additionally, the studies performed by various researchers highlight the necessity for ongoing examination and enhancement of sediment delivery and routing algorithms within SWAT. This entails investigating sensitive inputs, considering potential algorithm modifications, and exploring alternative approaches for sediment delivery. Moreover, there is a pressing need to improve routing methods to effectively consider diverse landscape elements such as depressions, riparian areas, and other distinctive features associated with specific landscapes [40]. Despite conducting sensitivity analyses or autocalibration of specific subsets of SWAT inputs in several studies, explicit uncertainty analysis results are missing. This underscores the need for more comprehensive uncertainty analyses in future applications of SWAT, considering the substantial uncertainty observed in many of the studies [40]. Further, ensuring that calibrated modeling results in SWAT accurately represent the real hydrologic and pollutant transport processes is crucial. Unrealistic calibration efforts, resulting from excessive adjustments of model parameters, are recognized as significant challenges in calibration and validation. Furthermore, there is a need for further research to improve and broaden influential statistical evaluation criteria [40].

Moreover, understanding the effect of spatial data resolution on model simulations is essential to enhance efficiency while maintaining accuracy. The selection of input data resolution in watershed models should align with the desired output. Collecting more detailed input data is advised to minimize uncertainties in predictions, given the growing reliance on models for watershed response forecasts. Besides, robust models can assess future climate scenarios and evaluate flood and drought risks [41,217]. In addition, SWAT, initially developed in the USA, was tailored to the soil data and land cover characteristics of that particular region. Consequently, researchers utilizing the model in different areas encounter difficulties in achieving precise simulation and analysis for their respective research locations. To overcome this challenge, it is essential for researchers to acquire and incorporate comprehensive databases of soil characteristics and land cover specific to their study areas into the model. This integration facilitates enhanced accuracy and applicability of the SWAT model in diverse geographical contexts [43]. Moreover, when it comes to watershed modeling in the Mediterranean region, there are inherent challenges due to limited availability of data required for the setup and calibration of the SWAT model. Nevertheless, empirical evidence indicates that the SWAT model has proven to be a valuable tool for addressing scientific, economic, and environmental concerns within the dynamic Mediterranean area, thereby suggesting the need for further investigations utilizing SWAT in Mediterranean watersheds [45]. Furthermore, in order to accurately capture the intricate heterogeneity and non-linear characteristics of karst flow and storage mechanisms, it is imperative to enhance the existing SWAT codes through additional advancements in future research [47]. It is crucial for upcoming studies to prioritize the development of solute transport models that effectively incorporate the diverse components and flow dynamics inherent in karst hydro-systems [47].

Furthermore, SWAT has demonstrated good performance in simulating coastal hydrology, but it is important to explore alternative models based on specific research goals and data accessibility [48]. A notable drawback of SWAT in coastal watershed modeling is its inability to replicate the two-way hydrodynamic tidal flow. To overcome this limitation, SWAT is often combined with a hydrodynamic model. The development of a user-friendly method to integrate SWAT with hydrodynamic models and accurately simulate tidal effects holds the potential to improve the effectiveness and applicability of SWAT in coastal watershed studies [48].

Due to the advancement of computer aid models, the integration of machine learning models for unsupervised classification and their impact on the accuracy of SWAT hydrological based models has shown an essential interest in the recent research trend. The main merit of the machine learning models offers the potential to improve the accuracy of land cover classification, a key input for hydrological modeling in SWAT. In comparison with the classical classification methods, they can provide the feasibility to extract the complex features from input data, enabling a more detailed and accurate land cover classification map. This enhanced classification can positively influence the estimation of crucial parameters in the SWAT model, such as vegetation characteristics and soil properties, thereby enhancing the accuracy of the model's simulations and predictions.

The capacity of the implications of machine learning can be further distinguished itself to address the potential of reducing the misclassification errors. On the contrary, classical classification methods may introduce errors when recognizable between land cover classes, particularly in cases of spectral or spatial overlaps. These misclassification errors can subsequently affect the parameterization of the SWAT model, leading to inaccurate simulations. Through the capacity of the machine learning models in capturing the complex patterns and relationships in the data, the risk of misclassification errors can be mitigated. The increased capacity of neural networks to differentiate between land cover classes can contribute to more precise parameter estimation in the SWAT model, thereby improving the accuracy of its outputs.

## 7 Conclusion

Despite the huge number of studies on SWAT applications in recent decades, detailed knowledge of the spatial and temporal resolution effects of model inputs is still restricted. The prediction of streamflow and sediment yield in a watershed is often subject to some degree of uncertainty owing to changes in the input data resolution. Obtaining the optimum resolution and accuracy that yields the best values of streamflow and sediment yield reduces the time, effort, and cost required to conduct comprehensive hydrological studies. This study provided a comprehensive overview of the spatial and temporal resolution of SWAT inputs and identified the best alternative climate product. The main findings indicated a scarcity of studies that dealt with the impact of the spatial and temporal resolution of SWAT inputs in large watersheds. There is a consensus that SWAT is not significantly sensitive to the finer DEM resolutions to estimate the surface runoff. In contrast, SWAT is genuinely sensitive to the finer DEM resolutions to estimate the sediment yield, and the 30 m DEM resolution provided high accuracy in sediment yield estimation in comparison with other coarser DEM resolutions. It is ultimately up to the watershed response and specific study area to determine which input DEM resolution should be used. STATSGO soil data slightly performed better than SSURGO soil data in simulating the streamflow and sediment yield before calibration, while SSURGO performed better after the calibration. On the other hand, setting up and calibrating a model needs time, effort, and computational resources, particularly in large watersheds. Therefore, the findings showed that less detailed soil data may be utilized instead of more detailed soil data, paying attention to the accuracy of soil data in the case of focusing on sediment yield production. Actually, studies related to soil data require significant investment, so publicly available data is often utilized instead. In contrast to studies that dealt with the effect of DEM resolution, which were thorough and detailed with satisfactory results, the literature has not paid enough attention to the influence of spatial resolution of LULC data on hydrological modeling, as their results were ambiguous, especially with regard to surface runoff accuracy. It is possible that this research scope will develop in the future. The sediment yield accuracy is highly dependent on the fine-resolution of the LULC dataset. This is due to the fact that CN is evaluated based on LULC, mean slope, and soil types within a basin. It could be concluded that more emphasis should be given to the LULC resolution than the DEM resolution.

Furtyer, the hourly rainfall resolution for SWAT simulation is promising for predicting streamflow more accurately, especially on days with heavy rainfall and longer rainfall hours. This review revealed that smaller precipitation time steps have produced a high amount of sediment yield. In soils that are less permeable, like clayey or loamy soils, and in watersheds with short travel times that are prone to surface runoff, small precipitation time steps may be beneficial. In lowland areas, the small precipitation time steps have little effect on model performance. Alternative climate products that have been identified in this review encompass four main categories: satellite precipitation products; gauge-based gridded data; climate reanalysis datasets; and ground-based weather radar. CFSR and TRMM are the most commonly utilized among the other climate products evaluated by SWAT modelers, followed by CHIRPS, CMADS, and PERSIANN-CDR. In the majority of the studies, CHIRPS consistently outperformed the other alternative inputs to SWAT in estimating the streamflow in watersheds with limited gauged data. The CFSR, on the other hand, performed poorly when compared to other products evaluated in several studies, where it tended to overestimate streamflow based on SWAT simulation.

Making predictions of spatial and temporal resolutions for each given study can be time-consuming. Therefore, a comprehensive summary and evaluation of the extent to which the accuracy of SWAT inputs affects the outputs has been provided to achieve optimal accuracy of the desired results.

## Availability of data and materials

Data sharing not applicable to this article as no datasets were generated or analyzed during the current study. It is a survey for the previous works.

## Funding

This research was funded by the Deanship of Research Oversight and Coordination (DROC), King Fahd University of Petroleum and Minerals, Dhahran, Saudi Arabia.

## CRediT authorship contribution statement

**Nisreen Jawad Rasheed:** Writing – review & editing, Writing – original draft, Visualization, Validation, Software, Resources, Methodology, Investigation, Formal analysis, Data curation, Conceptualization. **Mahmoud S. Al-Khafaji:** Writing – review & editing, Writing – original draft, Visualization, Validation, Supervision, Investigation, Formal analysis, Data curation, Conceptualization. **Imzahim A. Alwan:** Writing – review & editing, Writing – original draft, Visualization, Validation, Supervision, Investigation, Formal analysis, Data curation, Conceptualization. **Mohammad Saleh Al-Suwaiyan:** Writing – review & editing, Writing – original draft, Visualization, Validation, Supervision, Investigation, Formal analysis, Data curation, Conceptualization. **Ziaul Haq Doost:** Writing – review & editing, Writing – original draft, Visualization, Validation, Investigation. **Zaher Mundher Yaseen:** Writing – review & editing, Writing – original draft, Visualization, Validation, Supervision, Investigation, Formal analysis, Data curation, Conceptualization.

## Declaration of competing interest

The authors declare that they have no known competing financial interests or personal relationships that could have appeared to influence the work reported in this paper.
